# Minimal Residual Disease Diagnostics and Chimerism in the Post-Transplant Period in Acute Myeloid Leukemia

**DOI:** 10.1100/tsw.2011.16

**Published:** 2011-02-03

**Authors:** Ulrike Bacher, Torsten Haferlach, Boris Fehse, Susanne Schnittger, Nicolaus Kröger

**Affiliations:** ^1^ Department for Stem Cell Transplantation, University of Hamburg-Eppendorf, Germany; ^2^ MLL Munich Leukemia Laboratory, Munich, Germany

**Keywords:** chimerism, minimal residual disease (MRD), relapse, post-transplant period, acute myeloid leukemia (AML)

## Abstract

In acute myeloid leukemia (AML), the selection of poor-risk patients for allogeneic hematopoietic stem cell transplantation (HSCT) is associated with rather high post-transplant relapse rates. As immunotherapeutic intervention is considered to be more effective before the cytomorphologic manifestation of relapse, post-transplant monitoring gains increasing attention in stem cell recipients with a previous diagnosis of AML. Different methods for detection of chimerism (e.g., microsatellite analysis or quantitative real-time PCR) are available to quantify the ratio of donor and recipient cells in the post-transplant period. Various studies demonstrated the potential use of mixed chimerism kinetics to predict relapse of the AML. CD34^+^-specific chimerism is associated with a higher specificity of chimerism analysis. Nevertheless, a decrease of donor cells can have other causes as well. Therefore, efforts continue to introduce minimal residual disease (MRD) monitoring based on molecular mutations in the post-transplant period. The *NPM1* (nucleophosmin) mutations can be monitored by sensitive quantitative real-time PCR in subsets of stem cell recipients with AML, but for approximately 20% of patients, suitable molecular mutations for post-transplant MRD monitoring are not available so far. This emphasizes the need for an expansion of the panel of MRD markers in the transplant setting.

## INTRODUCTION

In acute myeloid leukemia (AML), allogeneic hematopoietic stem cell transplantation (HSCT) provides the strongest antileukemic effect. Due to the toxicity of allogeneic HSCT with the risk of infection, organ failure, or severe graft vs. host disease (GvHD), only patients with an adverse prognosis are selected for allogeneic HSCT, while those with a more favorable prognosis undergo standard treatment with chemotherapy only. Therefore, only those patients who are refractory to chemotherapy or at a high relapse risk due to unfavorable cytogenetic or molecular genetic profiles are candidates for allogeneic HSCT[1,2]. This selection of high-risk patients for allogeneic HSCT is associated with high relapse rates in the post-transplant period ranging from 20 to 60%, depending on parameters such as the genetic risk profiles or the intensity of conditioning.

Therapeutic options in the case of post-transplant relapse in AML patients may be based on immunotherapeutic approaches: withdrawal of immunosuppression or donor lymphocyte infusion (DLI)[3]. As these approaches are more effective when the leukemia cell load is smaller, attention focuses on a most early detection of post-transplant relapse. Two principles can be used.

Monitoring of chimerism means the assessment of the ratio of donor and recipient cells. This can be done by different PCR techniques[4,5,6] or interphase fluorescence *in situ* hybridization (FISH) in the case of sex-mismatched transplantation. Monitoring of chimerism is independent from the genetic AML subtypes. On the other hand, a decrease of donor cells is not necessarily associated with relapse of the underlying disease[7,8]. Restriction of chimerism analysis to defined cell populations, e.g., peripheral circulating $CD34^+$ precursor cells, may increase the specificity of method, but is laborious as additional cell sorting is required[9,10]. So far, standards for the intervals of measurement and for interpretation of chimerism results are missing, and thresholds for immunotherapeutic intervention are still individualized.

An alternative is measurement of the minimal residual disease (MRD) load in the post-transplant period. The highest sensitivity is offered by molecular techniques, e.g., quantitative real-time (RQ) PCR or nested PCR. Follow-up diagnostics based on molecular mutations guarantee higher specificity when compared to the monitoring of donor chimerism, but suitable molecular markers are only available for part of the AML patients, e.g., for patients with reciprocal gene fusions or with mutations of the *NPM1* (nucleophosmin) gene[11]. Only a few studies thus far have explored the potential of MRD monitoring specifically in the post-transplant period[11,12]. Immunophenotyping with multiparameter flow cytometry (MFC) can be universally performed in patients with AML[13,14], but provides lower sensitivity than the molecular methods. Only very few studies have been performed in the post-transplant period so far. This review summarizes the current status of post-transplant surveillance strategies for AML patients in the post-transplant period.

## GENETIC SUBGROUPS IN AML

AML is characterized by considerable genetic heterogeneity: 55% of all patients have cytogenetic abnormalities, such as the t(15;17)(q22;q12)/*PML-RARA* (in acute promyelocytic leukemia), the t(8;21)(q22;q22)/*RUNX1-RUNX1T1* (=*AML1-ETO*), or the inv(16)(p13q22)/t(16;16)(p13;q22)/*CBFB-MYH11* rearrangements. These genetic alterations confer a more favorable prognosis. In contrast, the prognosis of patients with 11q23/*MLL* rearrangements is adverse, with the exception of the t(9;11)(p22;q23). Various other cytogenetic alterations, e.g., of chromosomes 5, 7, or complex aberrant karyotypes (defined by the coincidence of more than three clonal cytogenetic abnormalities), also mediate an adverse prognosis[15]. Forty-five percent of all AML patients show no cytogenetic abnormalities and represent the large cytogenetic subgroup of “normal karyotype AML” with an intermediate prognosis. Molecular screening identifies genetic alterations in >85% of all normal karyotype AML cases[16] and is able to perform a prognostically relevant risk categorization, even in the absence of cytogenetic alterations. Most frequent are mutations of the *NPM1* gene (55% of all normal karyotype cases compared to 30% in overall AML)[17,18]. This mutation inhibits the normal shuttle function of the nucleophosmin protein between the nucleus and the cytoplasm, therefore impairing the normal function of the protein within the ARF-*P53* tumor-suppressor pathway. When compared to other AML subtypes, outcomes of patients with isolated *NPM1* mutations are more favorable. However, coincidence with the *FLT3* mutations is frequent and is associated with an adverse prognosis[19,20,21].

Internal tandem duplications of the *FLT3* gene (*FLT3*-ITDs) are found in 35–45% of all normal karyotype cases in AML. They lead to autophosphorylation of the *FLT3* receptor tyrosine kinase, and result in increased activation of signaling pathways and, finally, in enhanced cell proliferation[22]. The

*FLT3*-ITD mutations consist of insertions of variable length and are heterogeneously localized in the region that encodes the juxtamembrane domain of the *FLT3* receptor[23]. Carriers of an *FLT3*-ITD have higher rates of chemotherapy failure and relapse[24,25,26]. Alterations in the tyrosine kinase domain of the *FLT3* gene (*FLT3*-TKD) are represented by small nucleotide base exchanges. They are less frequent than the *FLT3*-ITD and probably have a weaker prognostic impact. Besides *NPM1* and *FLT3* mutations, a broad panel of molecular markers is detectable in AML, e.g., the prognostically adverse intragenic mutations of the *MLL* gene (*MLL*-PTD)[27] or mutations of *CEBPA* genes, which are considered to be prognostically favorable when they are biallelic and are found without concomitant adverse genetic markers[28].

## TECHNIQUES

### Chimerism

For the determination of chimerism (the ratio of donor and recipient alleles), Southern blot–based techniques (e.g., restriction-length fragment polymorphisms), which require a large quantity of DNA and are relatively insensitive, have been abandoned in favor of PCR techniques. Most centers perform microsatellite analysis of short tandem repeats (STR) by PCR[29], which provides qualitative or semi-quantitative assessment[30]. Separation of single leukocyte subsets after flow (FACS) or magnet-activated cell sorting (MACS) is able to improve the sensitivity of chimerism [10]. For gender-mismatch transplantations, RQ-PCR has been introduced on the basis of donor- and recipient-specific genetic polymorphisms or Y-specific sequences. This methodology guarantees high sensitivity of $10^{-4}$ to $10^{-5}$[4,31,32]. Interphase FISH for sex chromosomes provides an alternative[33,34]. A state of 100% hematopoiesis from donor origin is called “complete chimerism”, while the coexistence of donor and recipient hematopoiesis is called “mixed chimerism”[35]. For characterization of the kinetics of mixed chimerism, the categories “increasing mixed chimerism” (increase of recipient cells) and “decreasing mixed chimerism”, meaning a reduction of the percentages of recipient alleles or a switch from mixed to complete donor chimerism, have been introduced[36].

### Minimal Residual Disease (MRD)

Molecular monitoring of the residual leukemic cells below the microscopic level has been intensively studied for molecular mutations in the conservative treatment setting. RQ-PCR performs exact measurement of the amount of the PCR product in the sample by detection of fluorescent dyes or fluorescent-tagged DNA probes. Nested PCR is performed with two consecutive PCR reactions and two different primer pairs, both covering the region of interest. This results in highest sensitivity ($\sim10^{-6}$) and specificity. Immunophenotyping by MFC allows characterization of antigens on the cell surface or in the cytoplasm by use of monoclonal antibodies. Sensitivities of $10^{-3}$ to $10^{-4}$ are achieved. Leukemia-associated immunophenotypes (LAIPs) based on aberrant coexpression of antigens, aberrant fluorescence intensity of antigen expression, or lack of antigens on the leukemic cells can be detected in virtually every AML patient[37]. Considering the heterogeneity of patients with AML from genetic and phenotypic aspects, diagnostic algorithms for the post-transplant period should be individualized based on molecular mutation analysis and the LAIP as determined at diagnosis of the disease.

### Chimerism Studies in AML

In AML, various studies have suggested that the kinetics of chimerism is reliable for monitoring in the later post-transplant period. Bader et al.[38] performed semi-quantitative chimerism with STR marker amplification in 81 pediatric patients with AML. Increasing mixed chimerism – meaning an increase of recipient alleles – was detected in 19 cases. Despite early immunological intervention, the relapse rate was significantly higher in patients with increasing mixed chimerism than in patients with complete, low-level, or decreasing mixed chimerism (47 vs. 13%). The probability of 3-year event-free survival (EFS) was ~60% for patients with complete donor or decreasing mixed chimerism, but only <30% for patients with increasing mixed chimerism. In the case of increasing mixed chimerism, early immunological intervention significantly improved the probability for EFS compared to patients without intervention (36 vs. 0%)[38].

In contrast, Huisman et al.[39], who monitored chimerism by STR-based PCR in T- and non–T-cell subsets in 96 patients with AML after myeloablative or reduced-intensity conditioning, saw relapse preceded by increasing mixed chimerism in 25% of the cases only. Thus, although complete donor or decreasing mixed chimerism was significantly associated with a lower risk of associated relapse in the first 6 months post-transplant when compared to increasing mixed chimerism (31 vs. 83%), prediction of relapse was not reliable in this study[39].

The performance of chimerism after enrichment of $CD34^+$ cells may increase the reliability of chimerism measurement in myeloid malignancies. Zeiser et al.[40] performed both unseparated and lineage-specific chimerism analysis with the STR method in 168 patients with AML or myelodysplastic syndromes (MDS) after myeloablative conditioning. In those 41% of patients who developed recurrence of the malignancy, mixed chimerism was detected at a median of 28 days before clinical relapse by investigation of the unseparated samples and at a median of 30 days with $CD34^+$ specific chimerism. Patients with mixed chimerism as assessed by the $CD34^+$ technique had a relapse rate of 89%, while those with complete donor chimerism showed relapses in 6% of cases only[40]. Thiede et al.[9] explored chimerism in circulating $CD34^+$ cells after immunomagnetic pre-enrichment in 87 patients with different hematological disorders; of those, 28 with AML and six with MDS. A hematological relapse was observed in 20 patients in the $CD34^+$ fraction. In 14 of 16 patients, the donor chimerism decreased in the $CD34^+$ cells with a median of 52 days (range: 12–97 days) before the clinical manifestation of relapse[9].

In conclusion, the majority of studies were able to demonstrate significant correlations between the kinetics of increasing mixed chimerism and the clinical manifestation of relapse of AML. However, the intervals between the increase of recipient cells and the manifestation of relapse were short even when $CD34^+$ cells were investigated separately and relapse was not necessarily preceded by a decrease of donor cells. Recommendations of the different transplant centers with regards to the intervals of chimerism studies are variable; some recommend weekly performance, others recommend monthly monitoring.

## Introduction of the *NPM1* and *FLT3* Mutations in Post-Transplant Monitoring in AML?

Regarding MRD parameters in AML, measurement of the favorable reciprocal gene rearrangements – t(15;17)/*PML-RARA*, inv(16)/*CBFB-MYH11*, and t(8;21)/*RUNX1-RUNX1T1* – with RQ-PCR and nested PCR has been standardized in the chemotherapy setting[41,42,43]. Also in the post-transplant period, clearance of the *CBFB-MYH11* fusion transcript seemed to be strongly associated with sustained remission[44]. However, these favorable genetic alterations are rare in the transplant setting as chemotherapy alone is able to cure these AML subtypes in the majority of cases. Recent studies suggest that MRD monitoring can be done as well by RQ-PCR for the *NPM1* mutations that occur in ~50% of patients with normal karyotype AML. These mutations are prognostically favorable when they occur as isolated genetic alterations, but are often combined with the adverse *FLT3*-ITD gene[19,20,21]. Schnittger et al.[11] demonstrated high stability of the *NPM1* mutations in paired samples from 84 mutation carriers at relapse of the AML, meaning that the mutations were detectable again at the time point of relapse. Even in patients with relapses as late as 8 years from HSCT, the *NPM1* mutations seemed to be stable[45].

In the post-transplant setting, the number of patients investigated is very limited. In 13 patients carrying an *NPM1* mutation (subtype A), the achievement of molecular negativity after HSCT was the precondition for stable remissions, and an increase of the *NPM1* mutation level was detectable in 90% of patients before the manifestation of relapse[12]. Although the frequency of the *NPM1* mutation will be lower in the transplant than in the conventional treatment setting, it is assumed that RQ-PCR for this marker might soon enter post-transplant monitoring schedules for this subgroup.

The *FLT3*-ITDs are controversial with respect to post-transplant monitoring, as limited stability at relapse was reported[46]. In contrast, others demonstrated loss of the mutation in <5% of relapse cases[47]. Larger observational studies targeting the post-transplant period are missing so far. In four stem cell recipients who were carriers of the *FLT3*-ITD or -TKD before HSCT, Scholl et al. demonstrated strong correlations of the clinical courses with presence or absence of the mutation as determined with RQ-PCR[48]. Considering that RQ-PCR provides higher sensitivity than conventional PCR with gel electrophoresis or sequencing, the approach from Scholl et al. deserves attention. However, the labor intensity of the design of specific primers for the heterogeneous *FLT3*-ITD has to be seen, as these mutations are highly variable in size and localization.

Other recurrent molecular markers in AML, e.g., the *CEBPA* mutations[49] or the prognostically adverse *MLL*-PTD (partial tandem duplications of the *MLL* gene)[27], have not been evaluated in the post-transplant setting. Thus, MRD strategies based on molecular mutations are only available for subgroups of patients in the post-transplant setting. As PCR monitoring of molecular markers in the post-transplant period is closely linked to MRD analysis after conservative treatment, post-transplant surveillance of patients with AML will certainly have a benefit from an expansion of MRD markers in the conservative therapy setting. Similarly, results from MRD studies in AML patients post-transplant might be transferred to the conservative treatment setting.

## Quantification of *WT1* Gene Expression: a Subgroup-Spanning, but Less Specific Approach in AML

Expression of the *WT1* (Wilms tumor suppressor gene) was suggested to represent a reliable follow-up marker in AML[50] that can be performed across the borders of different genetic subgroups[51,52]. Also, for post-transplant monitoring, expression of the respective gene was investigated with RQ-PCR. Having analyzed 50 patients with AML in the post-transplant period, Ogawa et al. determined a distinct threshold that was able to predict relapse within 40 days in 100% of patients[53]. Sensitivity of *WT1* measurement with RQ-PCR ranges as high as $10^{-4}$, but the physiological background expression in healthy persons has to be considered. A comparison of *WT1* expression and *NPM1* mutation levels in 24 AML patients in the standard treatment setting by Barragan et al. demonstrated superiority of MRD monitoring for the *NPM1* mutations[54]. In contrast to *WT1*, which maintained residual expression levels in the period of complete remission, *NPM1* was undetectable in patients in stable remission and thus provided more safety. Therefore, it seems not very probable that *WT1* expression should be able to substitute measurement of chimerism.

## Multiparameter Flow Cytometry: a Subgroup-Spanning Approach for Post-Transplant Monitoring in AML?

With respect to immunophenotyping with MFC, LAIPs can be determined in principally all patients with AML. In the standard treatment scenario, monitoring of the amount of cells carrying the specific LAIP was shown to correlate significantly with the clinical courses[55,56], but only few data are available in the post-transplant setting. In a study from Perez-Simon et al. in 13 stem cell recipients with AML or MDS, persistence of LAIP-positive cells within the first 3–6 weeks post-transplant was associated with a relapse risk close to 60%, whereas absence of LAIP-positive cells was associated with stable remissions[57]. Diez-Campelo et al. performed MRD monitoring with MFC in 41 stem cell recipients with AML or MDS. A cut-off level of $10^{-3}$ leukemia cells 3 months post-transplant allowed them to discriminate patients with a 4-year EFS >70 vs. <20%[58]. Therefore, immunophenotyping seems able to contribute to monitoring in the post-transplant period, but an introduction of this approach in post-transplant routine requires additional studies (e.g., regarding the stability of LAIPs).

### Comparison of Peripheral Blood and Bone Marrow for MRD Monitoring in the Post-Transplant Period

Recently, RQ-PCR monitoring for the *NPM1* mutations (subtype A) was compared from peripheral blood and bone marrow in 25 stem cell recipients with *NPM1*-mutated AML. There was a high rate of concordant results as 74% of sample pairs were either MRD positive or negative in both materials. However, in 26% of all sample pairs, we observed weakly positive mutation levels in the bone marrow in contrast to negative results in the peripheral blood[59]. Thus, MRD monitoring for the *NPM1* mutations can be done by RQ-PCR from peripheral blood, which allows a higher frequency of analyses in the post-transplant period, but should be combined with investigation of bone marrow in regular intervals. Further studies should investigate this issue.

### Post-Transplant MRD Monitoring in Patients with Acute Lymphoblastic Leukemia (ALL)

In ALL, diverse studies investigated the potential of MRD techniques for post-transplant surveillance. Spinelli et al.[60] followed the *BCR-ABL1* and *MLL-AF4* gene fusions or rearrangements of the T-cell receptor or immunoglobulin genes in 43 adults with ALL by RQ-PCR. Patients with PCR negativity at day +100 post-transplant had a low relapse rate in contrast to those who were PCR positive (7 vs. 80%; $p = 0.0006$). Therefore, the authors concluded that MRD clearance may help to identify patients at high risk of leukemia relapse after HSCT who could benefit from pre-emptive treatments, such as donor lymphocyte infusion or new experimental drugs[60]. In a study from Wassmann et al.[61], 27 patients with Philadelphia-positive ALL received imatinib upon detection of MRD after HSCT (24 allogeneic, three autologous). Complete molecular remission was defined by complete clearance of transcripts with RQ-PCR and nested PCR in peripheral blood and bone marrow lasting for at least 3 months. All patients achieving MRD negativity early after the start of post-transplant imatinib remained in remission for the duration of tyrosine kinase inhibitor (TKI) therapy, while 12 of 13 patients who failed to achieve PCR negativity shortly after the start of imatinib developed relapses within a few months[61]. Earlier, Radich et al. investigated 36 patients with Philadelphia chromosome–positive ALL for the presence of the *BCR-ABL1* fusion mRNA transcript after allogeneic related/unrelated (n = 30), syngeneic (n = 1), or autologous HSCT (n = 5). Ten of 23 patients with at least one positive PCR result after HSCT relapsed between 28 and 416 days from the first positive MRD result. In contrast, only two relapses occurred in the 13 patients with no prior positive PCR assays[62].

Thus, in patients with ALL, post-transplant MRD testing has been successfully established and provides a valuable tool, e.g., for guidance of TKI therapy in this period.

## CONCLUSIONS

In the past 2 decades, clinical research managed to reduce transplant-related mortality (TRM) in acute leukemia patients, e.g., by improved supportive strategies or anti-infective therapy. Now, research focuses increasingly on the value of post-transplant monitoring strategies in myeloid malignancies[63], considering the high relapse risk that is the consequence of to the selection of poor-risk patients for allogeneic HSCT. Previously, MRD monitoring of patients with myeloid malignancies in the post-transplant period had been limited to chronic myeloid leukemia (CML), with measurement for residual *BCR-ABL1* transcript levels, which has been the guideline for adoptive immunotherapy by donor lymphocyte infusion for many years. In AML, chimerism is validated as best for post-transplant monitoring. Nevertheless, as the interpretation of chimerism results remains difficult in many cases, monitoring of the residual leukemic cell load based on mutations provides higher specificity. Only recently, MRD monitoring for the *NPM1* mutations was introduced in post-transplant surveillance strategies in patients with AML. This showed that distinct genetic subgroups of patients with AML can have a benefit from MRD strategies post-transplant. Immunophenotyping also has to be further validated for the post-transplant period in patients with AML.
